# Teratopyrones A–C, Dimeric Naphtho-γ-Pyrones and Other Metabolites from *Teratosphaeria* sp. AK1128, a Fungal Endophyte of *Equisetum arvense*

**DOI:** 10.3390/molecules25215058

**Published:** 2020-10-30

**Authors:** Ya-Ming Xu, A. Elizabeth Arnold, Jana M. U′Ren, Li-Jiang Xuan, Wen-Qiong Wang, A. A. Leslie Gunatilaka

**Affiliations:** 1 Southwest Center for Natural Products Research, School of Natural Resources and the Environment, College of Agriculture and Life Sciences, University of Arizona, 250 E, Valencia Road, Tucson, AZ 85706, USA; yamingx@arizona.edu; 2 School of Plant Sciences, College of Agriculture and Life Sciences, University of Arizona, Tucson, AZ 85721, USA; arnold@ag.arizona.edu (A.E.A.); juren@email.arizona.edu (J.M.U.); 3 State Key Laboratory of Drug Research, Shanghai Institute of Materia Medica, Chinese Academy of Sciences, 501 Haike Road, Zhangjiang Hi-Tech Park, Shanghai 201203, China; ljxuan@simm.ac.cn (L.-J.X.); wenqiong1019@126.com (W.-Q.W.)

**Keywords:** endophytic fungus, *Teratosphaeria*, teratopyrones, naphtho-γ-pyrones, nigerasperone A

## Abstract

Bioassay-guided fractionation of a cytotoxic extract derived from a solid potato dextrose agar (PDA) culture of *Teratosphaeria* sp. AK1128, a fungal endophyte of *Equisetum arvense*, afforded three new naphtho-γ-pyrone dimers, teratopyrones A–C (**1**–**3**), together with five known naphtho-γ-pyrones, aurasperone B (**4**), aurasperone C (**5**), aurasperone F (**6**), nigerasperone A (**7**), and fonsecin B (**8**), and two known diketopiperazines, asperazine (**9**) and isorugulosuvine (**10**). The structures of **1**–**3** were determined on the basis of their spectroscopic data. Cytotoxicity assay revealed that nigerasperone A (**7**) was moderately active against the cancer cell lines PC-3M (human metastatic prostate cancer), NCI-H460 (human non-small cell lung cancer), SF-268 (human CNS glioma), and MCF-7 (human breast cancer), with IC_50_s ranging from 2.37 to 4.12 μM while other metabolites exhibited no cytotoxic activity up to a concentration of 5.0 μM.

## 1. Introduction

Fungal endophytes constitute an abundant and underexplored group of fungi that inhabit plants in diverse natural and human-managed ecosystems [1,2]. These symbiotic fungi often produce bioactive metabolites, some of which may improve the growth or resiliency of the host plant [3]. Recent studies have demonstrated that fungal endophytes are rich sources of small-molecule natural products with novel structures and biomedical potential [4,5]. In our continuing search for bioactive and/or novel metabolites from endosymbiotic microorganisms [6], we have investigated a cytotoxic extract of the fungal endophyte, *Teratosphaeria* sp. AK1128, isolated from a photosynthetic stem of *Equisetum arvense* (field horsetail, Equisetaceae). Herein, we report cytotoxicity assay-guided fractionation of this extract, resulting in isolation and characterization of ten metabolites, including three new naphtho-γ-pyrone dimers, teratopyrones A–C (**1**–**3**), and the constituent responsible for cytotoxic activity, nigerasperone A (**7**). *Teratosphaeria* is a genus within the newly established fungal family Teratosphaeriaceae (Dothideomycetes, Ascomycota), which has been distinguished recently from *Mycosphaerella* (Mycosphaerellaceae) [7]. To the best of our knowledge, this constitutes the second report on metabolites of a fungal strain of the family Teratosphaeriaceae [8].

## 2. Results and Discussion

**Figure 1:**
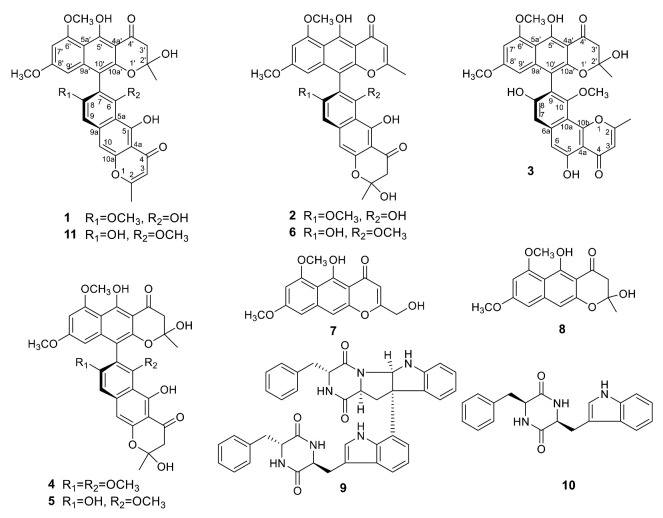
Structures of metabolites isolated from *Teratosphaeria* sp. AK1128.

The EtOAc extract of a PDA (potato dextrose agar) culture of *Teratosphaeria* sp. AK1128 exhibiting cytotoxic activity, on bioassy-guided fractionation involving solvent-solvent partitioning, Sephadex LH-20 size-exclusion and silica gel chromatography followed by HPLC purification, afforded metabolites **1**–**10** (Figure 1). Of these, **4**–**10** were previously known and were identified as naphtho-γ-pyrones, aurasperone B (**4**) [9], aurasperone C (**5**) [10], aurasperone F (**6**) [11], nigerasperone A (**7**) [12], and fonsecin B (**8**) [9], and diketopiperazines, asperazine (**9**) [13] and isorugulosuvine (**10**) [14], by comparison of their spectroscopic (^1^H NMR, ^13^C NMR, and LR-MS) data with those reported.

Spectroscopic (^1^H and ^13^C NMR, HRESIMS, and UV) data of teratopyrones A–C (**1**–**3**), together with their common molecular formula, C_31_H_26_O_11_, suggested that they are dimeric naphtho-γ-pyrones [11]. In their ^1^H and ^13^C NMR spectra, two sets of signals were observed in different intensity ratios and this was suspected to be due to the atropisomerism around the C_7_–C_10′_ axis [10] and/or the presence of C-2 and C-2′ hemi-ketal stereoisomeric mixtures as a result of non-enzymatic formation of this moiety during the biosynthesis of naphtho-γ-pyrones [15]. Although several recent publications on dimeric naphtho-γ-pyrones report only one set of the NMR data, careful examination of the spectra of these dimeric naphtho-γ-pyrones provided in the Supporting Information of these papers indicated the presence of two sets of signals, corresponding to two possible tautomers [16,17,18]. The atropisomerization around the C_7_–C_10′_ axis is known to be restricted under mild conditions [10], and the atropisomer ratio for a given dimeric naphtho-γ-pyrone may depend on its producer fungus [19]. Although it is not possible to obtain the atropisomer ratio directly, the use circular dichroism (CD) data for the assignment of stereochemistry of the binaphthyl moiety of the major atropisomer of dimeric naphtho-γ-pyrones by the exciton chirality method has been reported [20,21].

**Figure 2:**
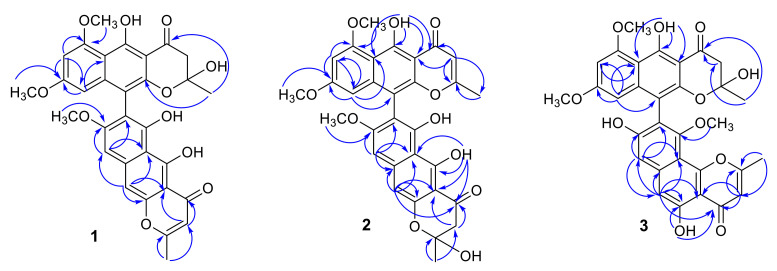
Selected HMBC correlations of teratopyrones A–C (**1**–**3**).

**Figure 3:**
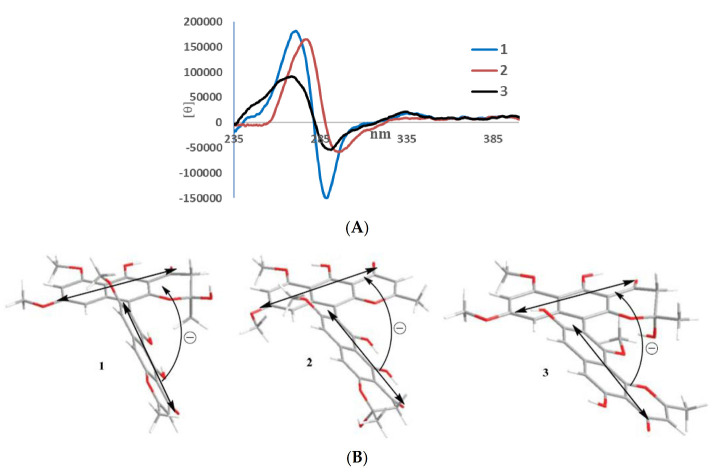
ECD spectra (**A**) and negative exciton chirality (**B**) of teratopyrones A–C (**1**–**3**).

In its ^1^H NMR spectrum, teratopyrone A (**1**) exhibited signals due to two methyls (δ_H_ 1.43 (s) and 2.36 (s)), three methoxyls (δ_H_ 3.39 (s), 3.60 (s), and 3.90 (s)), five aromatic protons (δ_H_ 5.99 (s), 6.12 (br. s), 6.26 (br. s), 7.06 (s), and 7.07 (s)), and two hydrogen-bonded phenolic hydroxy groups (δ_H_ 14.48 (s) and 14.97 (s)). The ^13^C NMR spectrum of **1** (Table 1) contained 31 carbon signals due to its major tautomer and these were assigned with the help of HSQC and HMBC spectra. The two carbonyl signals at δ_C_ 197.9 and 184.6 revealed that **1** was made up of two naphtho-γ-pyrone monomers, of which one was hydrated at C_2′_–C_3′_. The HMBC data for **1** (Figure 2) revealed that the linkage of two monomers of **1** was the same as that of nigerasperone C (**11**; Figure 1)) in which the hydrated monomer consisted of the upper unit [12]. The ^13^C NMR data of **1** closely resembled those of **11** [12], except for the signals in the vicinity of C-5–C-8. However, some differences were observed for C-5 (δ_C_ 162.5 for **1**; 166.1 for **11**), C-6 (δ_C_ 107.1 for **1**; 111.8 for **11**), and C-8 (δ_C_ 157.3 for **1**; 163.1 for **11**), suggesting that **1** differed from **11** in the attachment of hydroxy/methoxy groups at C-6/C-8. The attachment of the methoxy group to C-8 in **1** was confirmed by the HMBC correlations of 8-OCH_3_ (δ_H_ 3.38) and H-9 (δ_H_ 7.06) to C-8 (δ_C_ 157.3). The ECD spectrum of **1** (Figure 3) showed Davydov-split Cotton effects as negative ([θ] −1.46 × 10^5^, 289.5 nm) first and positive ([θ] +1.78 × 10^5^, 272 nm) second, suggesting negative chirality [21] and M-configuration of the 7′–10′ bond [20]. Thus, the structure of teratopyrone A was determined as (10′*S*)-2′,5,5′′,6-tetrahydroxy-6′,8,8′-trimethoxy-2,2′-dimethyl-2′,3′-dihydro-4*H*,4′*H*-[7,10′-bibenzo[g] chromene]-4,4′-dione (**1**) (Figure 1).

Teratopyrone B (**2**) also exhibited UV adsorption bands at 202, 238, 281.5, and 386 nm typical for naphtho-γ-pyrones [11]. Similar to **1**, the ^1^H NMR spectrum of **2** in DMSO-d_6_ solution showed two sets of signals due to the presence of two tautomeric forms in almost equal amounts (10:8 ratio) and these consisted of two methyl singlets (δ_H_ 1.65 (1.64 for the other conformer) and 2.55), three methoxy singlets (δ_H_ 3.41 (3.40), 3.60 (3.59), and 4.00), five aromatic signals (δ_H_ 6.19 (6.17), 6.55, 6.61 (2H), and 6.90), and two singlets due to hydrogen-bonded phenolic hydroxy groups (δ_H_ 14.27 (14.25), and 13.19 (13.18)). The ^13^C NMR spectrum of **2** (Table 1) resembled closely that of aurasperone F (**6**). The ^13^C NMR signals belonging to each of the conformers of **2** were recognized and assigned with the help of HSQC and HMBC data. The presence of two carbonyl signals at δ_C_ 198.4 and 182.3 indicated that **2** is a dimer consisting of a naphtho-γ-pyrone monomer and its hydrated form. The positions of attachment of OCH_3_ groups in **2** were determined by the analysis of its HMBC spectrum (Figure 2). The presence of 8-OCH_3_ in **2** was confirmed by the HMBC correlations of H-9 (δ_H_ 6.90) to C-8 (δ_C_ 159.3) and 8-OCH_3_ (δ_H_ 4.00) to C-8 (δ_C_ 159.3). The ECD spectrum of **2** (Figure 3) showed Davydov-split Cotton effects as negative ([θ] −5.70 × 10^4^, 297 nm) first and positive ([θ] +1.63 × 10^5^, 278 nm) second, suggesting the negative chirality [21] and M-configuration of the 7–10′ bond [20]. Therefore, the structure teratopyrone B was determined as (10′*S*)-2,5,5′,6-tetrahydroxy-6′,8,8′-trimethoxy-2,2′-dimethyl-2,3-dihydro-4*H*,4′*H*-[7,10′-bibenzo[g]chromene]-4,4′-dione (**2**) (Figure 1), suggesting that it is the isomer of aurasperone F (**6**) with different methyl ether positions at C-6 and C-8.

The ^1^H NMR spectrum of teratopyrone C (**3**) also exhibited two sets of signals belonging to two tautomers in the ratio 3:2 as a result of the hemi-ketal tautomerism and consisted of two methyl singlets (δ_H_ 1.40 (1.78 for another tautomer) and 2.47), three methoxy singlets (δ_H_ 3.46 (3.53), 3.55, and 3.91 (3.89)), five aromatic proton singlets (δ_H_ 5.99 (5.95), 6.44 (6.39), 6.50, 6.93, and 7.00 (7.02)), and two hydrogen-bonded phenolic hydroxy singlets (δ_H_ 14.23 and 12.89 (12.88)). The hydrogen-bonded phenolic hydroxy singlet at δ_H_ 12.89 (12.88) suggested the existence of an angular naphtho-γ-pyrone moiety [9]. The ^13^C NMR spectrum of **3** (Table 1) contained signals due to each tautomer that were assigned separately with the help of HSQC and HMBC data. The two carbonyl signals at δ_C_ 198.4 and 182.4 (182.3) indicated that **3** consisted of one each of naphtho-γ-pyrone and hydrated naphtho-γ-pyrone monomers. The positions of attachment of OCH_3_ groups in **3** were also determined by the analysis of its HMBC spectrum (Figure 2). The HBMC correlation of 5-OH (δ_H_ 12.89) to C-6 (δ_C_ 104.3), further supporting the existence of angular naphtho-γ-pyrone moiety, and the correlations of H-7 (δ_H_ 7.01) to C-8 (δ_C_ 158.9), H-7 (δ_H_ 7.01) to C-9 (δ_C_ 117.9), and 10-OCH_3_ (δ_H_ 3.53 (3.46)) to C-8 (δ_C_ 156.8) established the C_9_–C_10′_ linkage and 8-methoxyl group. The ECD spectrum of **3** (Figure 3) exhibited Davydov-split Cotton effects as negative ([θ] −5.14 × 10^4^, 292 nm) first and positive ([θ] +8.82 × 10^4^, 278 nm) second, suggesting the negative chirality [21] and M-configuration of the 9–10′ bond [20]. Therefore, the structure of teratopyrone C was determined as 9-((10*R*)-2,5-dihydroxy-6,8-dimethoxy-2-methyl-4-oxo-3,4-dihydro-2*H*-benzo[g]chromen-10-yl)-5,8-dihydroxy-10-methoxy-2-methyl-4*H*-benzo[h]chromen-4-one (**3**) (Figure 1).

Previous studies have shown that some naphtha-γ-pyrone dimers such as chaetochromins exhibit cytotoxic activity [22]. Thus, teratopyrones A–C and other metabolites encountered were evaluated for their cytotoxic activity, employing a panel of five cancer cell lines and normal cells. Among these, only nigerasperone A (**7**) showed cytotoxic activity at a concentration <5.0 μM. The IC_50_s of **7** for these cancer cell lines were determined as 4.12 ± 0.32 μM (PC-3M (human metastatic prostate cancer)), 3.01 ± 0.11 μM (NCI-H460 (human non-small lung cancer)), 2.37 ± 0.15 μM (SF-268 (human central nervous system glioma)), 3.90 ± 0.33 μM (MCF-7 (human breast cancer)), and >5.0 μM for MDA-MB-231 (human metastatic breast cancer) and WI-38 (normal human lung fibroblast cells). Interestingly, nigerasperone A (**7**) has been reported previously to be inactive against A549 (human alveolar adenocarcinoma) and SMMC-7721 (human hepatocellular carcinoma) cancer cell lines [12]. These and our data suggest that nigerasperone A (**7**) may be selectively cytotoxic against some cancer cell lines. 

## 3. Materials and Methods

### 3.1. General Procedures

Optical rotations were measured with a Jasco Dip-370 polarimeter (JASCO Inc., Easton, MD, USA.) using CHCl_3_ as solvent. ECD spectra were measured with JASCO J-810 (JASCO Inc., Easton, MD, USA). First, 1D and 2D NMR spectra were recorded in CDCl_3_ or DMSO-d_6_ with a Bruker Avance III 400 instrument (Bruker BioSpin Corporation, San Jose, CA, USA) at 400 MHz for ^1^H NMR and 100 MHz for ^13^C NMR using residual CHCl_3_ as the internal standard. Chemical shift values (*δ*) are given in parts per million (ppm) and the coupling constants are in Hz. Low-resolution and high-resolution MS were recorded on Shimadzu LCMS-DQ8000α (Shimadzu Scientific Instruments, Inc., Columbia, MD, USA) and Agilent G6224A TOF mass spectrometers (Agilent Technologies Co. Ltd., Beijing, China), respectively. HPLC (Waters Corporation, Milford, MA, USA) was carried out on a 10 × 250 mm Phenomenex Luna 5μ C18 (2) column with Waters Delta Prep system consisting of a PDA 996 detector.

### 3.2. Fungal Isolation and Identification

In June 2008, a healthy individual of *Equisetum arvense* was collected from Beringian tundra in the Seward Peninsula of Western Alaska (64°30′04″ N, 165°24′23″ W; 6 m.a.s.l.) [23]. The photosynthetic stem was washed in tap water and cut into ca. 2 mm^2^ segments that were surface-sterilized by agitating sequentially in 95% EtOH for 30 s, 0.5% NaOCl for 2 min, and 70% EtOH for 2 min [23]. Forty-eight tissue segments were surface-dried under sterile conditions and then placed individually onto 2% malt extract agar (MEA) in sterile 1.5 mL micro centrifuge tubes. Tubes were sealed with Parafilm^TM^ and incubated under ambient light/dark conditions at room temperature (ca. 21.5 °C) for up to one year. Emergent fungi were isolated into pure culture on 2% MEA, vouchered in sterile water, and deposited as living vouchers at the Robert L. Gilbertson Mycological Herbarium at the University of Arizona. One fungus of interest, isolate AK1128, was used for the present study. This fungus has been deposited at the University of Arizona Robert L. Gilbertson Mycological Herbarium (accession number AK1128).

Total genomic DNA was isolated from fresh mycelium of the isolate AK1128 and the nuclear ribosomal internal transcribed spacers and 5.8s gene (ITS rDNA; ca. 600 base pairs (bp)) and an adjacent portion of the nuclear ribosomal large subunit (LSU rDNA; ca. 500 bp) was amplified as a single fragment by PCR. Positive amplicons were sequenced bidirectionally as described previously [23]. A consensus sequence was assembled and basecalls were made by *phred* [24] and *phrap* [25] with orchestration by Mesquite [26], followed by manual editing in Sequencher (Gene Codes Corp.). The resulting sequence was deposited in GenBank (accession JQ759476).

Because the isolate did not produce diagnostic fruiting structures in culture, two methods were used to tentatively identify isolate AK1128 via molecular sequence data. First, the LSU rDNA portion of the sequence was evaluated using the naïve Bayesian classifier for fungi [27] available through the Ribosomal Database Project (http://rdp.cme.msu.edu/). The Bayesian classifier estimated placement within the Capnodiales (Dothideomycetes) with high support, but placement at finer taxonomic levels was not possible. Therefore, the entire sequence was compared against the GenBank database using BLAST [28]. The top ten BLAST matches were to unidentified dothideomycetous endophytes or uncultured ascomycetous clones, except for two strains of *Colletogloeopsis dimorpha* (strains CBS 120085 and CBS 120086). The matches to *Colletogloeopsis dimorpha* had 97% coverage and a maximum identity of only 92%, and similar levels of match precision to other taxa restricted our taxonomic inference.

Therefore, to clarify the phylogenetic placement and taxonomic assignment of AK1128, two phylogenetic analyses were conducted. First, the top 99 BLAST matches were downloaded from GenBank, four problematic sequences for which quality was suspect were removed, and AK1128 and the resulting dataset was aligned automatically via MUSCLE (http://www.ebi.ac.uk/Tools/msa/muscle/) with default parameters. The alignment consisted of dothideomycetous endophytes as well as described species of Dothideomycetes that mostly comprised taxa affiliated with some lineages recognized within *Mycosphaerella* (e.g., *Teratosphaeria, Colletogloeopsis, Catulenostroma,* etc.). The alignment was trimmed so that starting and ending points were generally consistent with the sequence length for AK1128 and adjusted manually in Mesquite [26] prior to analysis. The final dataset consisted of 96 sequences and 1084 characters. This dataset was analyzed by maximum likelihood in GARLI (Zwickl, D. J. Genetic algorithm approaches for the phylogenetic analysis of large biological sequence datasets under the maximum likelihood criterion (Ph.D. Dissertation, The University of Texas at Austin, Austin, TX, USA, 2008) using the GTR + I + G model of evolution as determined by ModelTest [29]. The resulting topology indicated that AK1128 had an affinity for *Teratosphaeria, Catenulostroma*, and relatives but was not phylogenetically affiliated with *Colletogloeopsis* (data not shown). Because this analysis included many unknown taxa (endophytes) and we could not root the tree with certainty, the taxon sampling was found to be insufficient to infer with confidence the placement of the AK1128.

A second phylogenetic analysis was therefore conducted using taxa affiliated with *Teratosphaeria, Catulenostroma,* and relatives, as analyzed previously [30]. The alignment of ITS rDNA sequences from [30] was obtained from TreeBase and pruned to include only those taxa of interest based on our analysis above (i.e., the lower half of Figure 1 in [30] in a preliminary analysis, and then only those taxa most closely related within *Teratosphaeria* in the final analysis). The sequence for AK1128 was integrated into the pruned dataset and the data were realigned, adjusted, and analyzed as described above. The resulting dataset consisted of 79 sequences and 582 characters in the preliminary analysis. A bootstrap analysis with 1000 replicates was conducted to assess topological support and the topology was rooted with *Devriesia strelitziae* [30]. The resulting tree suggested that AK1128 is affiliated with *Teratosphaeria* species (data not shown).

Therefore, the dataset was pruned further, focusing only on those *Teratosphaeria* species suggested to be close relatives of AK1128. The final analysis included 42 terminal taxa and 554 characters and was rooted with *Catulenostroma macowanii* based on the topology of the preliminary analysis. The final tree with maximum likelihood bootstrap values is shown in Supplemental Figure S13. The final analysis placed AK1128 with certainty within the genus *Teratosphaeria* (Teratosphaeriaceae), likely in affiliation with *T. associata (*known from *Eucalyptus* [31] and *Protea* [30] from Australia). Given the distinctive geographical origin and host of AK1128, and the sister relationship of AK1128 to known strains of *T. associata* (Supplemental Figure S13), we designate the strain as *Teratosphaeria* sp. AK1128, affiliated with but distinct from known variants of *T. associata*.

### 3.3. Cultivation and Isolation of Metabolites

A culture of *Teratosphaeria* sp. AK1128 grown on PDA for two weeks was used for extraction. The PDA cultures from 20 T-flasks were combined and extracted with MeOH (5.0 L) in an ultrasonic bath for 1.0 h. After filtration, the MeOH solution was concentrated to around one-third of its volume in vacuo, and the resulting solution was extracted with EtOAc (3 × 700.0 mL). The EtOAc solution was concentrated in vacuo to afford the crude extract (1.223 g). The crude extract, which showed activity in cytotoxicity assay, was fractionated by solvent–solvent partitioning using 80% aq. MeOH (100.0 mL) and hexanes (3 × 100.0 mL), followed by 50% aq. MeOH (obtained from 80% aq. MeOH layer by adding calculated volume of water) and CHCl_3_ (3 × 100.0 mL) to afford hexanes, CHCl_3_, and 50% aq. MeOH fractions of which only the CHCl_3_ fraction was found to be cytotoxic. Therefore, the CHCl_3_ fraction (1.094 g) was subjected to gel-permeation chromatography on Sephadex LH-20 (50.0 g). The column was eluted with 250.0 mL each of 1:4 hexanes-CH_2_Cl_2_, 3:2 CH_2_Cl_2_-acetone, 1:4 CH_2_Cl_2_-acetone, and MeOH to give four fractions. The cytotoxic 3:2 CH_2_Cl_2_-acetone fraction (778.6 mg) was further fractionated by SiO_2_ gel (100.0 g) column chromatography using 95:5 CHCl_3_-MeOH as eluting solvent. Eight combined fractions (fractions 1–8) were obtained by combining the fractions based on their TLC profiles. Fraction 1 (12.5 mg) was separated by reversed-phase HPLC (70% aq. MeOH as eluant) to afford **1** (7.4 mg, t_R_ 16.5 min) and **8** (2.7 mg, t_R_ 10.5 min). Reversed-phase HPLC separation of a portion (55.6 mg) of fraction 2 (135.8 mg) using 65% aq. MeOH gave additional amounts of **8** (3.7 mg, t_R_ 14.4 min) and **4** (29.6 mg, t_R_ 25.6 min). Compounds **3** (16.9 mg, t_R_ 26.2 min), **4** (10.8 mg, t_R_ 18.1 min), and **7** (4.2 mg, t_R_ 15.5 min) were obtained from fraction 3 (70.8 mg) by reversed-phase HPLC using 65% aq. MeOH as the eluent. Fraction 4 (29.1 mg) was further purified by reversed-phase HPLC (65% aq. MeOH) to afford **2** (8.3 mg, t_R_ 27.7 min) and **6** (9.8 mg, t_R_ 21.6 min). A portion (78.5 mg) of fraction 5 (299.7 mg) was subjected to reversed-phase HPLC. Elution with 65% aq. MeOH yielded metabolites **5** (54.5 mg, t_R_ 15.3 min) and **6** (10.3 mg). Reversed-phase HPLC separation of fraction 6 (106.0 mg) using 60% aq. MeOH as eluent gave two crude fractions, A (t_R_ 10.8 min) and B (t_R_ 27.7 min). Further purification of fractions A and B by normal-phase SiO_2_ HPLC (eluent: 97.5:2.5 CHCl_3_-MeOH) afforded **10** (2.3 mg, t_R_ 15.0 min) and **9** (26.0 mg, t_R_ 17.5 min), respectively.

Teratopyrone A (**1**). Yellow amorphous solid; [α]${}_{D}^{25}$ −54.4 (c 0.1, CHCl_3_); UV (MeOH) *λ*_max_ (log *ε*) 203 (4.45), 229 (4.63), 281.5 (4.84), 403 (4.09); ECD (MeOH) [*θ*] +1.78 × 10^5^ (272 nm), −1.46 × 10^5^ (289.5 nm); ^1^H NMR (400 MHz, CDCl_3_) *δ* 14.97 (s, 1H, 5-OH), 14.48 (s, 1H, 5′-OH), 7.08/7.03 (br s, 1H, H-10), 7.07/7.04 (br s, 1H, H-9), 6.26/6.28 (br s, 1H, H-7′), 6.12 (br s, 1H, H-9′), 5.98 (s, 1H, H-3′), 3.90/3.94 (s, 3H, 6′-OMe), 3.60/3.59 (s, 3H, 8′-OMe), 3.39/3.55 (s, 3H, 8-OMe), 3.35 (m, 1H, H-3′), 2.80/2.81 (m, 1H, H-3′), 2.35 (s, 3H, 2-Me), 1.42/1.47 (s, 3H, 2′-Me); ^13^C NMR data, see Table 1; HRESIMS *m*/*z* 575.1546 [M + H]^+^ (calcd. for C_31_H_27_O_11_, 575.1548).

Teratopyrone B (**2**). Yellow amorphous solid; [α]${}_{D}^{25}$ +39.7 (*c* 0.105, CHCl_3_); UV (MeOH) *λ*_max_ (log *ε*) 202 (4.36), 238 (4.66), 281.5 (4.73), 386 (3.95); ECD (MeOH) [*θ*] + 1.63×10^5^ (278 nm), −5.70 × 10^4^ (297 nm); ^1^H NMR (400 MHz, DMSO-d_6_) *δ* 14.27/14.25 (s, 1H, 5-OH), 13.19/13.18 (s, 1H, 5′-OH), 6.90/6.85 (br s, 1H, H-9), 6.61 (br s, 2H, H-10, H-7′), 6.54 (s, 1H, H-3′), 6.19/6.17 (d, 1H, *J* = 2.0, H-9′), 4.00 (s, 3H, 8-OMe), 3.60/3.59 (s, 3H, 8′-OMe), 3.41/3.40 (s, 3H, 6′-OMe), 3.25/3.23 (d, 1H, *J* = 12.6, H-3′), 2.78/2.79 (d, 1H, *J* = 12.6, H-3′), 2.55 (s, 3H, 2′-Me), 1.65/1.64/1.69 (s, 3H, 2-Me); ^13^C NMR data, see Table 1; HRESIMS *m*/*z* 575.1543 [M + H]^+^ (calcd. for C_31_H_27_O_11_, 575.1548). 

Teratopyrone C (**3**). Yellow amorphous powder; [α]${}_{D}^{25}$ +19.3 (*c* 0.07, CHCl_3_); UV (MeOH) *λ*_max_ (log *ε*) 204 (4.52), 237.5 (4.80), 281 (4.80), 395 (4.03); ECD (MeOH) [*θ*] +8.82 × 10^4^ (278 nm), −5.14 × 10^4^ (292 nm); ^1^H NMR (400 MHz, DMSO-d_6_) *δ* 14.42 (s, 1H, 5′-OH), 12.89/12.88 (s, 1H, 5-OH), 7.00/7.02 (br s, 1H, H-7), 6.94 (br s, 1H, H-6), 6.50 (s, 1H, H-3), 6.44/6.39 (br s, 1H, H-7′), 5.99/5.95 (br s, 1H, H-9′) 3.91/3.89 (s, 3H, 6′-OMe), 3.55 (s, 3H, 8′-OMe), 3.46/3.53 (s, 3H, 10-OMe), 3.09 (m, 1H, H-3′), 2.86 (m, 1H, H-3′), 2.47 (s, 3H, 2-Me), 1.40/1.78 (s, 3H, 2′-Me); ^13^C NMR data, see Table 1; HRESIMS *m*/*z* 575.1551 [M + H]^+^ (calcd for C_31_H_27_O_11_, 575.1548).

### 3.4. Cytotoxicity Assay

A resazurin-based colorimetric (alamarBlue) assay was used for evaluating the cytotoxic activity of metabolites **1**–**10** against androgen-sensitive human prostate adenocarcinoma (LNCaP), metastatic human prostate adenocarcinoma (PC-3M), human breast (MCF-7), human non-small cell lung (NCI-H460), and human CNS glioma (SF-268) cancer cell lines and normal human lung fibroblast (WI-38) cells as described previously [32]. Doxorubicin and DMSO were used as positive and negative controls, respectively.

## 4. Conclusions

Ten metabolites, including three new dimeric naphtha-γ-pyrones, teratopyrones A–C (**1**–**3**), were isolated from a PDA culture of the endophytic fungus, *Teratosphaeria* sp. AK1128. This constitutes the second report of the occurrence of secondary metabolites in a fungus of the family Teratosphaeriaceae. The ECD spectra of teratopyrones A–C suggested that all three of them have negative exciton chirality. Cytotoxicity data for nigerasperone A (**7**) from this and previous studies suggest that it has selective activity for certain cancer cell lines.

## Figures and Tables

**Table 1:** ^13^C NMR data (100 MHz, *δ*) for teratopyrones A–C (**1**–**3**).

Position	1	2	3
	CDCl_3_	DMSO-d_6_	DMSO-d_6_
2	168.1 s	100.0 s	167.9/167.8 s
3	107.1 d	47.8 t	109.7 d
4	184.6 s	198.4 s	182.4/182.3 s
4a	104.2 s	103.0 s	107.9 s
5	162.5 s	163.0 4/163.98 s	155.2/155.0 s
5a (6) *	107.1 s	108.6 s	104.3 d
6 (6a) *	160.9 s	159.2 s	139.8 s
7	117.6 s	116.3 s	104.9 d
8	157.3 s	159.3 s	158.9 s
9	105.7 d	105.4 d	117.9 s
9a (10) *	142.3 s	142.2 s	156.9 s
10 (10a) *	101.2 d	101.4 d	106.6 s
10a (10b) *	152.8 s	153.3 s	154.8 s
2-Me	20.7 q	27.6 q	19.9 q
6-OMe			60.8 q
8-OMe	62.0 q	56.4 q	
2′	100.2 s	167.9/167.8 s	100.3 s
3′	46.7 t	109.8 d	48.4 t
4′	197.9 s	182.3 s	198.4 s
4a′	103.5 s	107.6 s	102.7 s
5′	162.5 s	153.7 s	163.9 5
5a′	107.1 s	109.7 s	106.2/106.4 s
6′	161.8 s	159.0 s	160.7/161.5 s
7	96.1 d	96.4/96.3 d	94.9/95.1 d
8′	162.5 s	161.0 s	161.3/161.7 s
9′	96.5 d	97.0/96.8 d	96.9/97.6 d
9a′	140.2 s	140.2 s	142.3 s
10′	103.1 s	104.1 s	105.4/105.5 s
10a′	153.3 s	155.0 s	154.7 s
2′-Me	27.8 q	20.1 q	27.6/26.8 q
6′-OMe	56.0 q	55.2/55.1 q	56.1 q
8′-OMe	55.2 q	61.2/61.4 q	55.0/54.9 q

* The position number in parentheses is for teratopyrone C (**3**).

## Supplementary Materials

The following are available online, Figure S1: ^1^H NMR Spectrum (400 MHz) of Teratopyrone A (1) in CDCl_3_, Figure S2. ^13^C NMR Spectrum (100 MHz) of Teratopyrone A (1) in CDCl_3_, Figure S3. HSQC Spectra (400 MHz) of Teratopyrone A (1) in CDCl_3_, Figure S4. HMBC Spectra (400 MHz) of Teratopyrone A (1) in CDCl_3_, Figure S5. ^1^H NMR Spectrum (400 MHz) of Teratopyrone B (2) in DMSO-d_6_, Figure S6. ^13^C NMR Spectrum (100 MHz) of Teratopyrone B (2) in DMSO-d_6_, Figure S7. HSQC Spectrum (400 MHz) of Teratopyrone B (2) in DMSO-d_6_, Figure S8. HMBC Spectrum (400 MHz) of Teratopyrone B (2) in DMSO-d_6_, Figure S9. ^1^H NMR Spectrum (400 MHz) of Teratopyrone C (3) in DMSO-d_6_, Figure S10. ^13^C NMR Spectrum (100 MHz) of Teratopyrone C (3) in DMSO-d_6_, Figure S11. HSQC Spectrum (400 MHz) of Teratopyrone C (3) in DMSO-d_6_, Figure S12. HMBC Spectrum (400 MHz) of Teratopyrone C (3) in DMSO-d_6_, Figure S13. Results of maximum likelihood analysis placing strain AK1128 within *Teratosphaeria* with high support.

## References

[1] Rodriguez R.J., White J.F., Arnold A.E., Redman R.S. (2009). Fungal endophytes: Diversity and functional roles: Tansley review. New Phytol..

[2] Higginbotham S.J., Arnold A.E., Ibañez A., Spadafora C., Coley P.D., Kursar T.A. (2013). Bioactivity of fungal endophytes as a function of endophyte taxonomy and the taxonomy and distribution of their host plants. PLoS ONE.

[3] Kusari S., Pandey S.V., Spiteller M. (2013). Untapped mutualistic paradigms linking host plant and endophytic fungal production of similar bioactive secondary metabolites. Phytochemistry.

[4] Gunatilaka A.A.L. (2006). Natural Products from Plant-associated Microorganisms: Distribution, Structural Diversity, Bioactivity, and Implications of Their Occurrence. J. Nat. Prod..

[5] Zhang H.W., Song Y.C., Tan R.X. (2006). Biology and chemistry of endophytes. Nat. Prod. Rep..

[6] Wijeratne E.M.K., Gunaherath G.M.K.B., Chapla V.M., Tillotson J., de la Cruz F., Kang M.-J., U’Ren J.M., Araujo A.R., Arnold A.E., Chapman E. (2016). Oxaspirol B with p97 inhibitory activity and other oxaspirols from *Lecythophora* sp. FL1375 and FL1031, endolichenic fungi inhabiting *Parmotrema tinctorum* and *Cladonia evansii*. J. Nat. Prod..

[7] Crous P.W., Braun U., Groenewald J.Z. (2007). Mycosphaerella is polyphyletic. Stud. Mycol..

[8] Padumadasa C., Xu Y.M., Wijeratne E.M.K., Espinosa-Artiles P., U’Ren J.M., Arnold A.E., Gunatilaka A.A.L. (2018). Cytotoxic and noncytotoxic metabolites from *Teratosphaeria* sp. FL2137, a fungus associated with *Pinus clausa*. J. Nat. Prod..

[9] Priestap H.A. (1984). New naphthopyrones from *Aspergillus fonsecaeus*. Tetrahedron.

[10] Tanaka H., Wang P.-L., Namiki M. (1972). Yellow pigments of *Aspergillus niger* and *A. awamori*. III. Structure of aurasperone C. Agric. Biol. Chem..

[11] Bouras N., Mathieu F., Coppel Y., Lebrihi A. (2005). Aurasperone F–a new member of the naphtho-gamma-pyrone class isolated from a cultured microfungus, *Aspergillus niger* C-433. Nat. Prod. Res..

[12] Zhang Y., Li X.M., Wang B.G. (2007). Nigerasperones A–C, new monomeric and dimeric naphtho-γ-pyrones from a marine alga-derived endophytic fungus *Aspergillus niger* EN-13. J. Antibiot..

[13] Varoglu M., Corbett T.H., Valeriote F.A., Crews P. (1997). Asperazine, a selective cytotoxic alkaloid from a sponge-derived culture of *Aspergillus niger*. J. Org. Chem..

[14] Tullberg M., Grotli M., Luthman K. (2006). Efficient synthesis of 2,5-diketopiperazines using microwave assisted heating. Tetrahedron.

[15] Fujii I., Watanabe A., Sankawa U., Ebizuka Y. (2001). Identification of Claisen cyclase domain in fungal polyketide synthase WA, a naphthopyrone synthase of *Aspergillus nidulans*. Chem. Biol..

[16] Shaaban M., Shaaban K.A., Abdel-Aziz M.S. (2012). Seven naphtho-γ-pyrones from the marine-derived fungus *Alternaria alternata*: Structure elucidation and biological properties. Org. Med. Chem. Lett..

[17] Siriwardane A., Kumar N.S., Jayasingh L., Fujimoto Y. (2015). Chemical investigation of metabolites produced by an endophytic *Aspergillus* sp. isolated from *Limonia acidissima*. Nat. Prod. Res..

[18] He Y., Tian J., Chen X., Sun W., Zhu H., Li Q., Lei L., Yao G., Xue Y., Wang J. (2016). Fungal naphtho-γ-pyrones: Potent antibiotics for drug-resistant microbial pathogens. Sci. Rep..

[19] Akiyama K., Teraguchi S., Hamasaki Y., Mori M., Tatsumi K., Ohnishi K., Hayashi H. (2003). New dimeric naphthopyrones from *Aspergillus niger*. J. Nat. Prod..

[20] Koyama K., Natori S., Iitaka Y. (1987). Absolute configurations of chaetochromin A and related bis(naphtho-γ-pyrone) mold metabolites. Chem. Pharm. Bull..

[21] Harada N., Nakanishi K. (1972). The exciton chirality method and its application to configurational and conformational studies of natural products. Acc. Chem. Res..

[22] Koyama K., Ominato K., Natori S., Tashiro T., Tsuruo T. (1988). Cytotoxicity and antitumor activities of fungal bis(naphtho-γ-pyrone) derivatives. J. Pharmacobiodyn..

[23] U′Ren J.M., Lutzoni F., Miadlikowska J., Laetsch A.D., Arnold A.E. (2012). Host and geographic structure of endophytic and endolichenic fungi at a continental scale. Am. J. Bot..

[24] Ewing B., Green P. (1998). Base-calling of automated sequencer traces using phred. II. Error probabilities. Genome Res..

[25] Ewing B., Hillier L., Wendl M.C., Green P. (1998). Base-calling of automated sequencer traces using phred. I. Accuracy assessment. Genome Res..

[26] Maddison W.P., Maddison D.R. (2009). Mesquite: A Modular System for Evolutionary Analysis, Version 2.6. http://mesquiteproject.org.

[27] Liu K.L., Porras-Alfaro A., Kuske C.R., Eichorst S.A., Xie G. (2011). Accurate, rapid taxonomic classification of fungal large-subunit rRNA genes. Appl. Environ. Microbiol..

[28] Altschul S.F., Gish W., Miller W., Myers E.W., Lipman D.J. (1990). Basic local alignment search tool. J. Mol. Biol..

[29] Posada D., Crandall K.A. (1998). MODELTEST: Testing the model of DNA substitution. Bioinformatics.

[30] Crous P.W., Summerell B.A., Mostert L., Groenewald J.Z. (2008). Host specificity and speciation of *Mycosphaerella* and *Teratosphaeria* species associated with leaf spots of Proteaceae. Persoonia.

[31] Crous P.W., Summerell B.A., Carnegie A.J., Mohammed C., Himaman W., Groenewald J.Z. (2007). Foliicolous *Mycosphaerella* spp. and their anamorphs on *Corymbia* and *Eucalyptus*. Fungal Divers..

[32] Wijeratne E.M.K., Bashyal B.P., Liu M.X., Rocha D.D., Gunaherath G.M.K.B., U’Ren J.M., Gunatilaka M.K., Arnold A.E., Whitesell L., Gunatilaka A.A.L. (2012). Geopyxins A-E, ent-kaurane diterpenoids from endolichenic fungal strains *Geopyxis aff. majalis* and *Geopyxis* sp. AZ0066: Structure–activity relationships of geopyxins and their analogues. J. Nat. Prod..

