# Supplementary material for: Teratopyrones A–C, Dimeric Naphtho-γ-Pyrones and Other Metabolites from Teratosphaeria sp. AK1128, a Fungal Endophyte of Equisetum arvense
Source: Molecules. 2020 Oct 30;25(21):5058. doi: 10.3390/molecules25215058 (PMC7662774; doi:10.3390/molecules25215058)

Supplementary Material for

Teratopyrones A–C, Dimeric Naphtho-γ-pyrones and Other Metabolites from *Teratosphaeria* sp. AK1128, a Fungal Endophyte of *Equisetum arvense*

Ya-ming Xu [1], A. Elizabeth Arnold [2], Jana M. U'Ren [2], Li-Jiang Xuan[3], Wen-qiong Wang[3], A. A. Leslie Gunatilaka [1,*]

[1] Natural Products Center, School of Natural Resources and the Environment,
College of Agriculture and Life Sciences, University of Arizona, 250 E. Valencia Road, Tucson, Arizona 85706, United States; yamingx@arizona.edu; leslieg1@email.arizona.edu

[2] School of Plant Sciences, College of Agriculture and Life Sciences, University of Arizona, Tucson, Arizona 85721, United States; juren@arizona.edu; barnoldaz@gmail.com

[3] State Key Laboratory of Drug Research, Shanghai Institute of Materia Medica, Chinese Academy of Sciences, 501 Haike Road, Zhangjiang Hi-Tech Park, Shanghai 201203, P. R. China; ljxuan@simm.ac.cn; wenqiong1019@126.com

* Correspondence: leslieg@cals.arizona.edu; Tel.: + (520) 621-9932

Table of Contents

Figure S1. ^{1}H NMR Spectrum (400 MHz) of Teratopyrone A (**1**) in $CDCl_3$ ……………………....……………………….. S3

Figure S2. ^{13}C NMR Spectrum (100 MHz) of Teratopyrone A (**1**) in $CDCl_3$ ………..………………………………… S4

Figure S3. HSQC Spectra (400 MHz) of Teratopyrone A (**1**) in $CDCl_3$ ………………..…………………………………… S5

Figure S4. HMBC Spectra (400 MHz) of Teratopyrone A (**1**) in $CDCl_3$ ……………………………………………………...S6

Figure S5. ^{1}H NMR Spectrum (400 MHz) of Teratopyrone B (**2**) in DMSO-d_6 ……………………………………………….. S7

Figure S6. ^{13}C NMR Spectrum (100 MHz) of Teratopyrone B (**2**) in DMSO-d_6………..………………………………..….. S8

Figure S7. HSQC Spectrum (400 MHz) of Teratopyrone B (**2**) in DMSO-d_6……………………………………………... S9

Figure S8. HMBC Spectrum (400 MHz) of Teratopyrone B (**2**) in DMSO-d_6…………………………………… …... S10

Figure S9. ^{1}H NMR Spectrum (400 MHz) of Teratopyrone C (**3**) in DMSO-d_6 ………………………………….… …. S11

Figure S10. ^{13}C NMR Spectrum (100 MHz) of Teratopyrone C (**3**) in DMSO-d_6 …………………………………… ……. S12

Figure S11. HSQC Spectrum (400 MHz) of Teratopyrone C (**3**) in DMSO-d_6………………………………………...……. S13

Figure S12. HMBC Spectrum (400 MHz) of Teratopyrone C (**3**) in DMSO-d_6 ………………………………………………. .S14

Figure S13. Results of maximum likelihood analysis placing strain AK1128 within *Teratosphaeria* with high support. Numbers in association with nodes indicate bootstrap values (values ≥ 70 are shown). ……………………………………...S15

Figure S1. ^{1}H NMR Spectrum (400 MHz) of Teratopyrone A (**1**) in $CDCl_3$

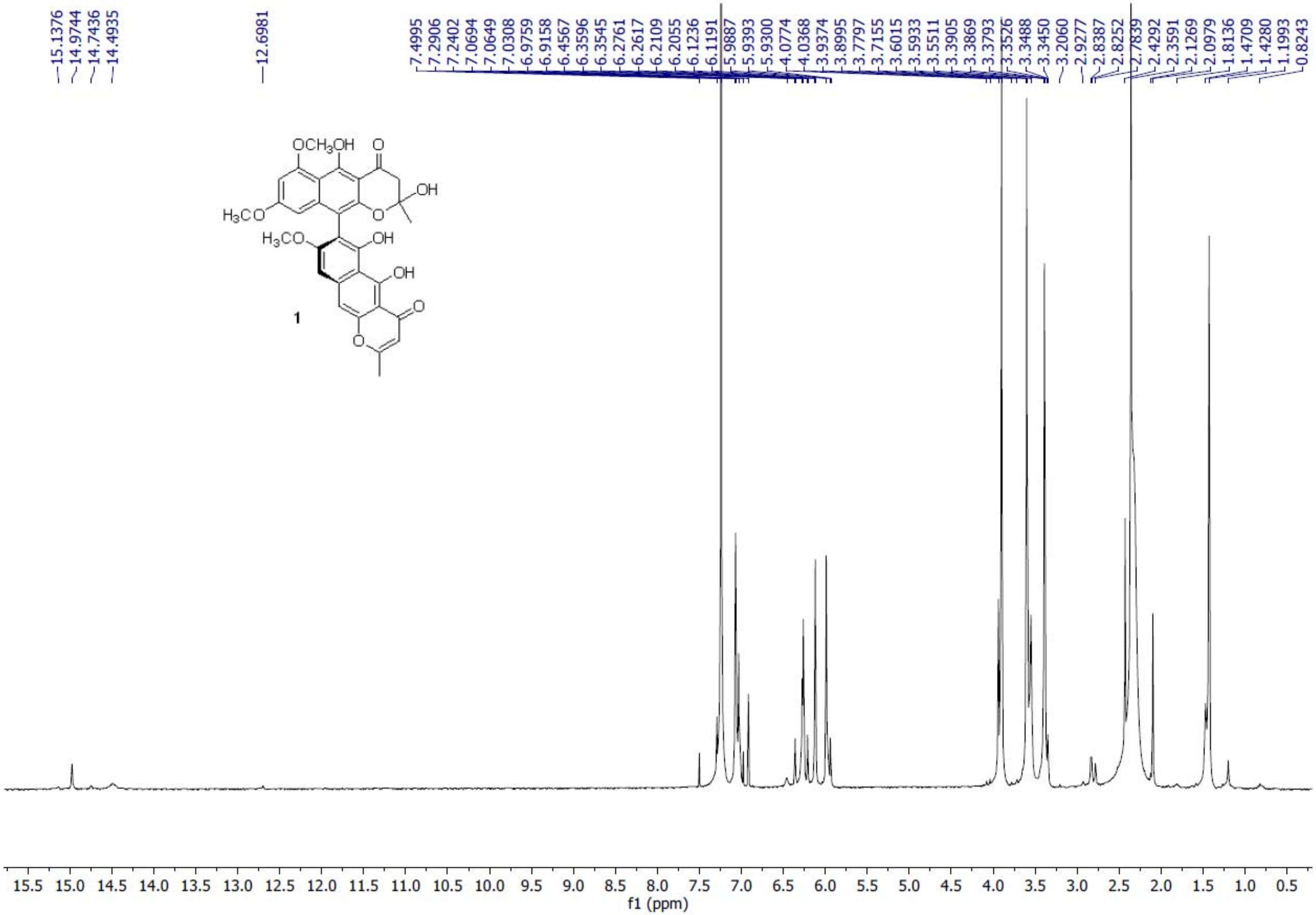

Figure S2. ^{13}C NMR Spectrum (100 MHz) of Teratopyrone A (**1**) in $CDCl_3$

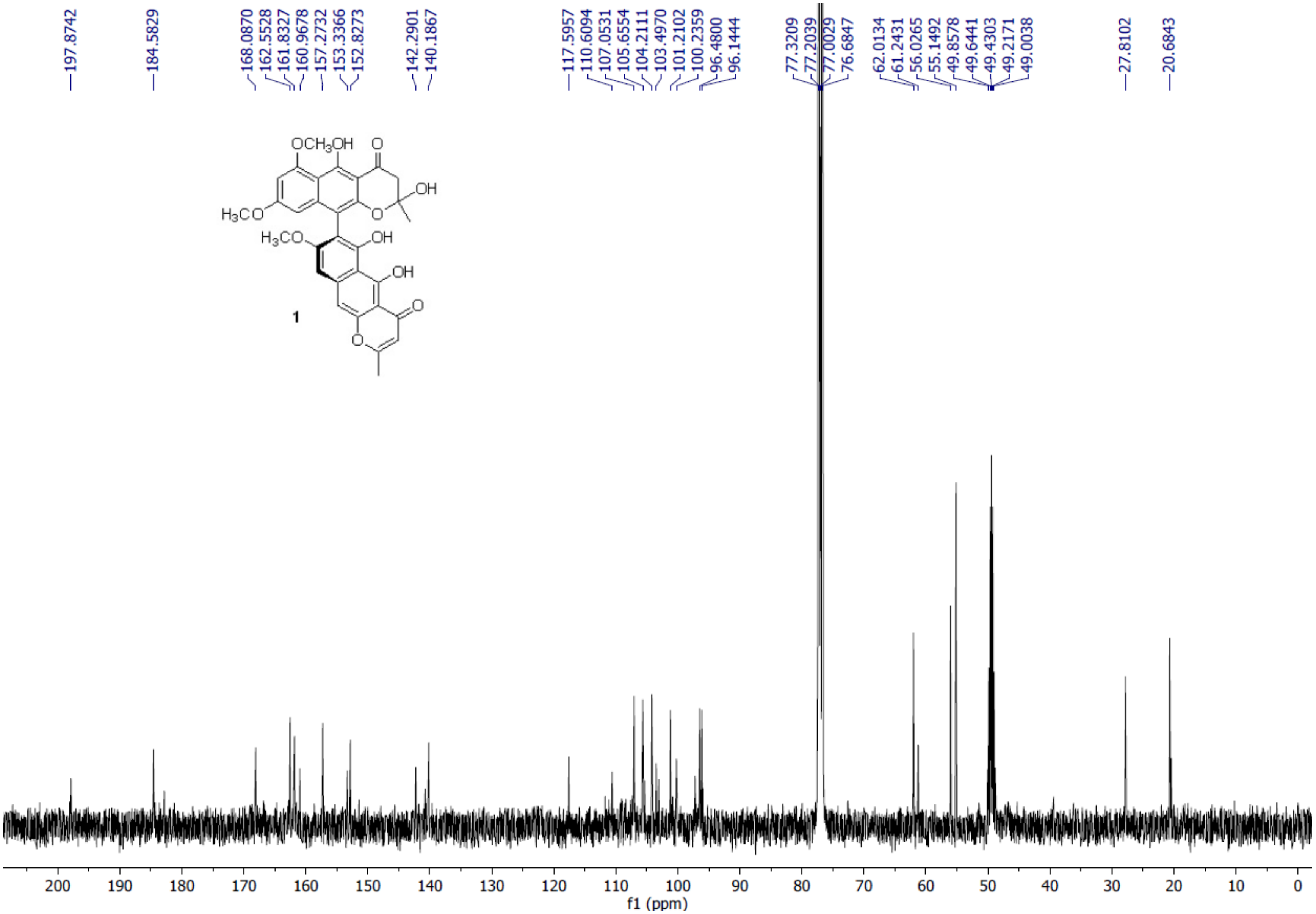

Figure S3. HSQC Spectra (400 MHz) of Teratopyrone A (**1**) in $CDCl_3$

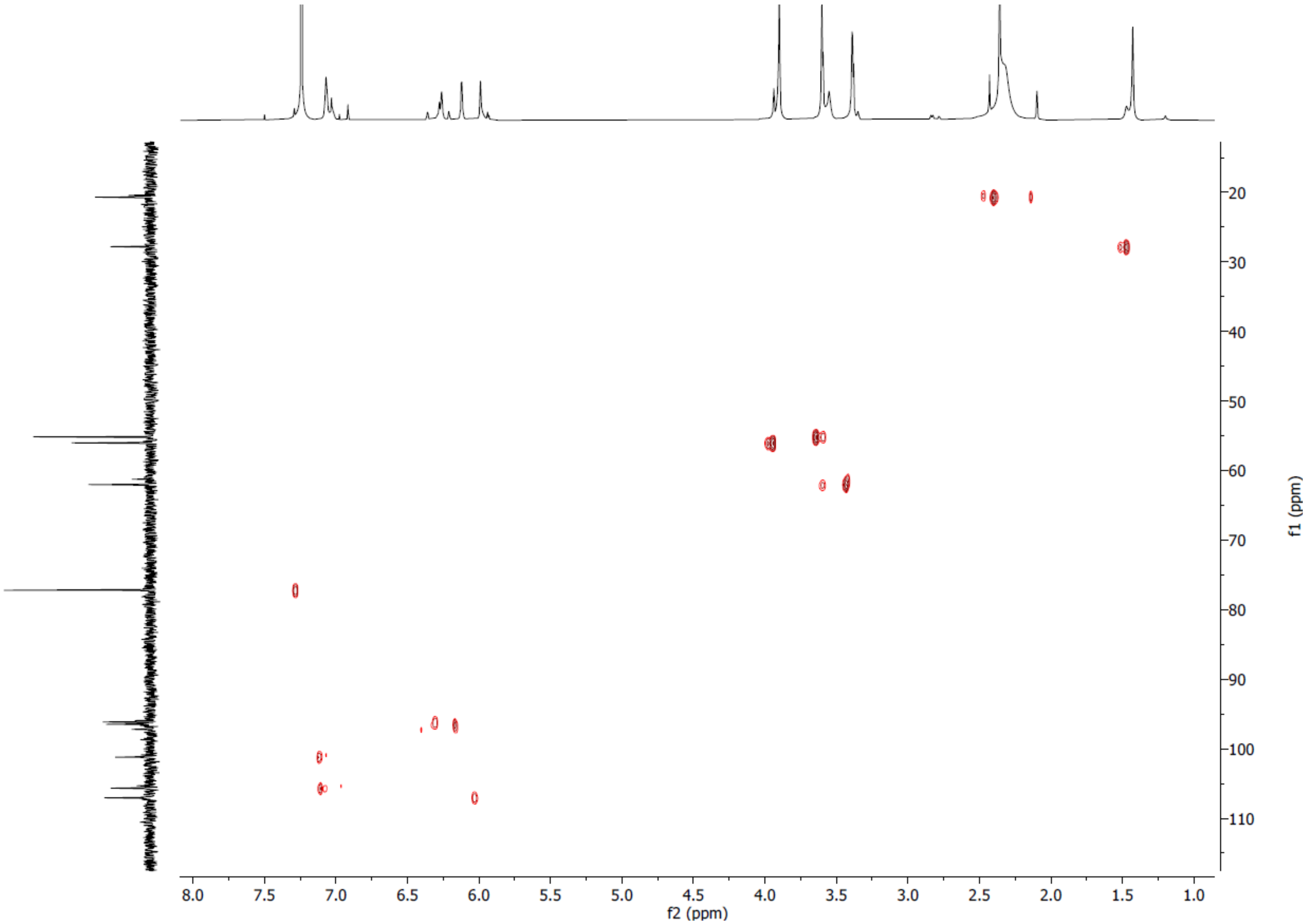

Figure S4. HMBC Spectra (400 MHz) of Teratopyrone A (**1**) in $CDCl_3$

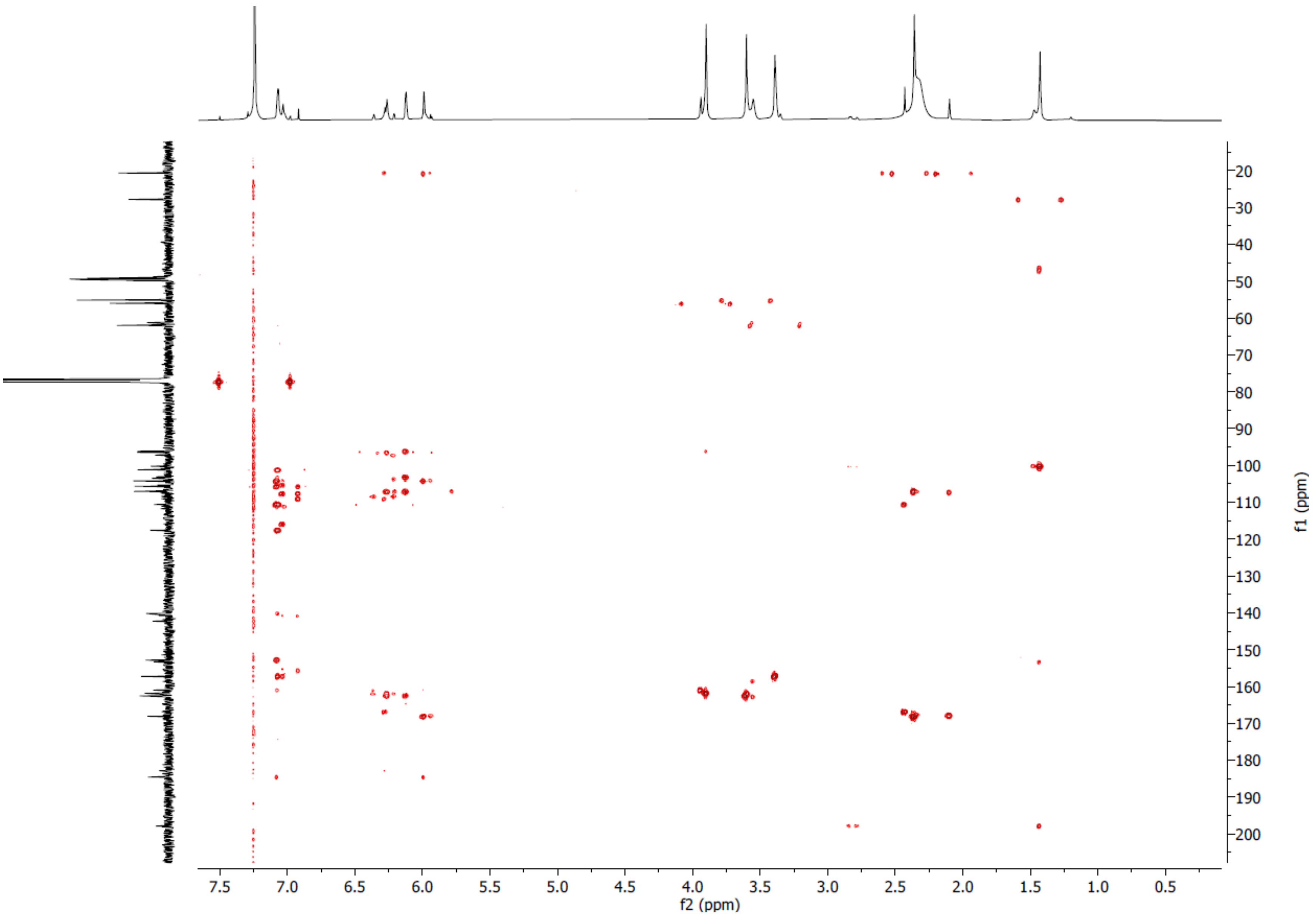

Figure S5. ^{1}H NMR Spectrum (400 MHz) of Teratopyrone B (**2**) in DMSO-d6

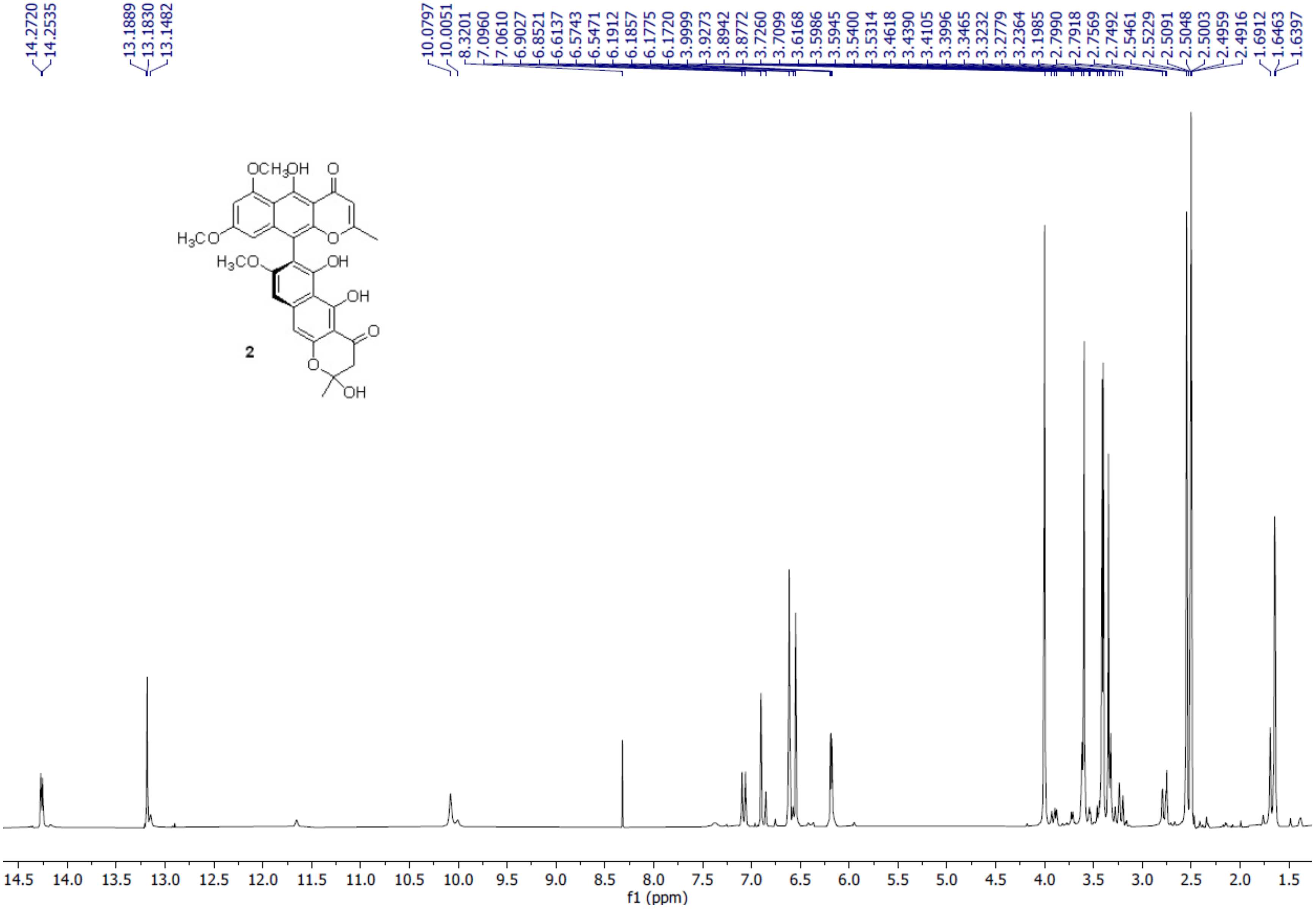

Figure S6. ^{13}C NMR Spectrum (400 MHz) of Teratopyrone B (**2**) in DMSO-d_6

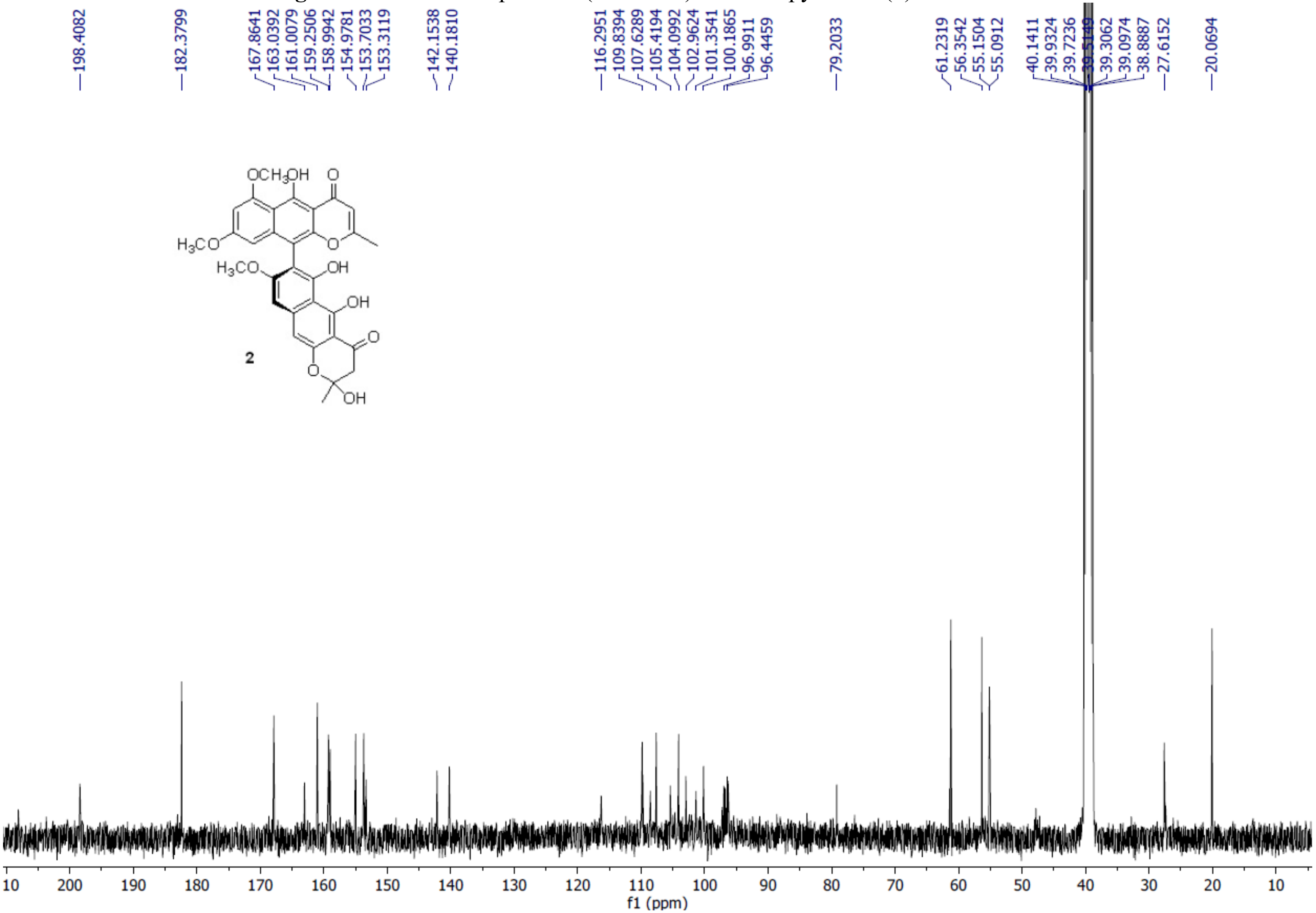

Figure S7. HSQC Spectrum (400 MHz) of Teratopyrone B (**2**) in DMSO-d_6

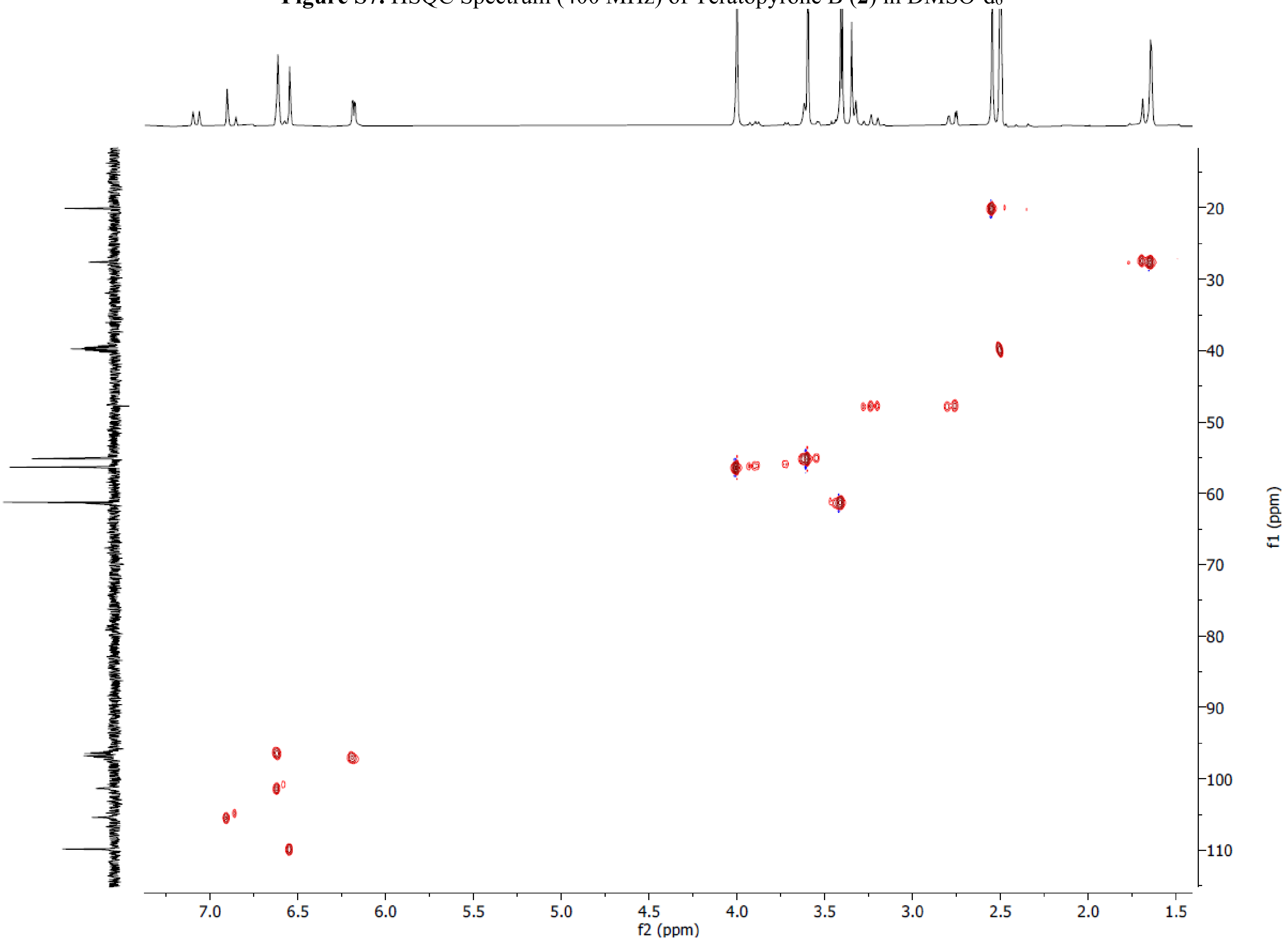

Figure S8. HMBC Spectrum (400 MHz) of Teratopyrone B (**2**) in DMSO-d6

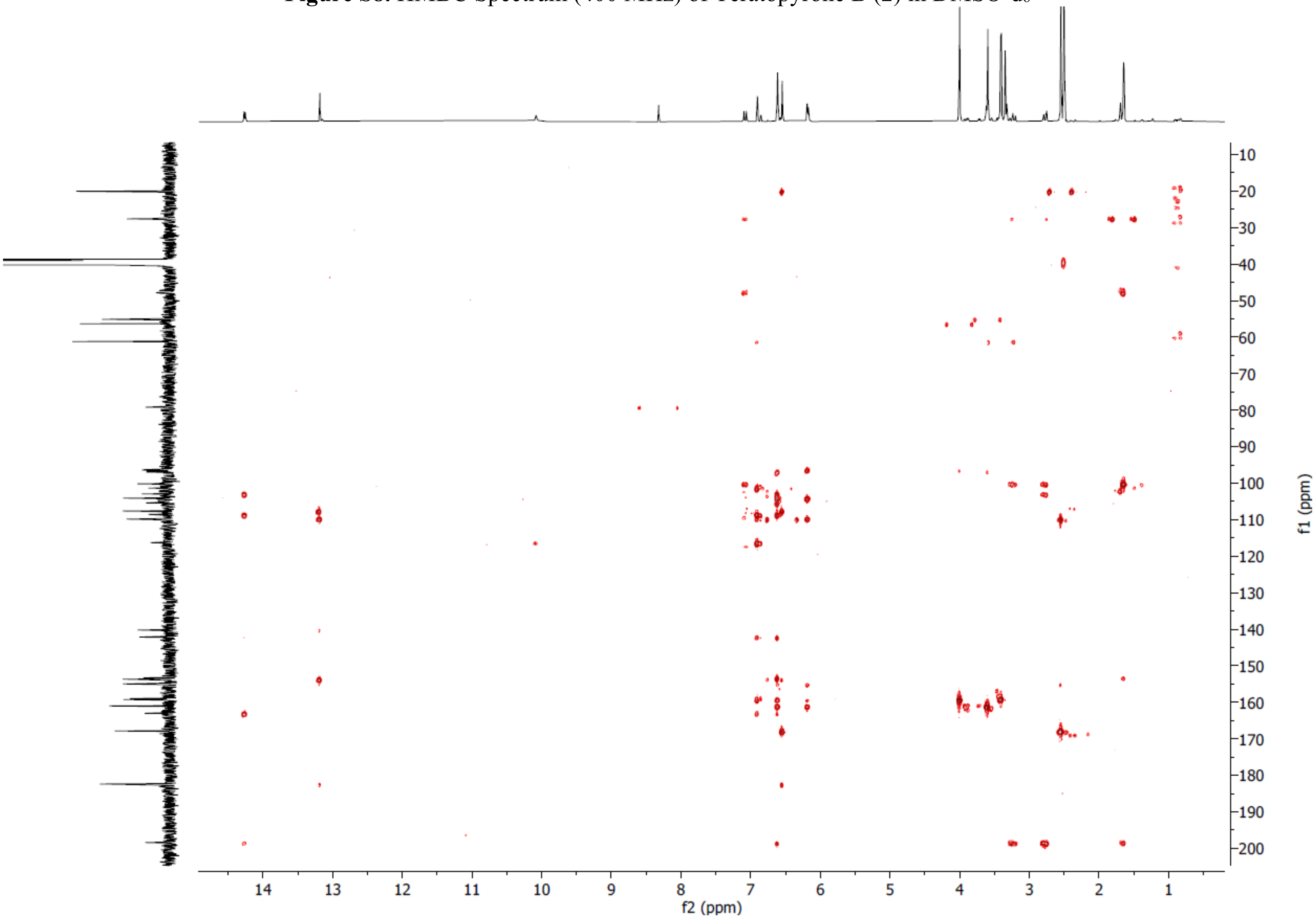

Figure S9. ^{1}H NMR Spectrum (400 MHz) of Teratopyrone C (**3**) in DMSO-d_6

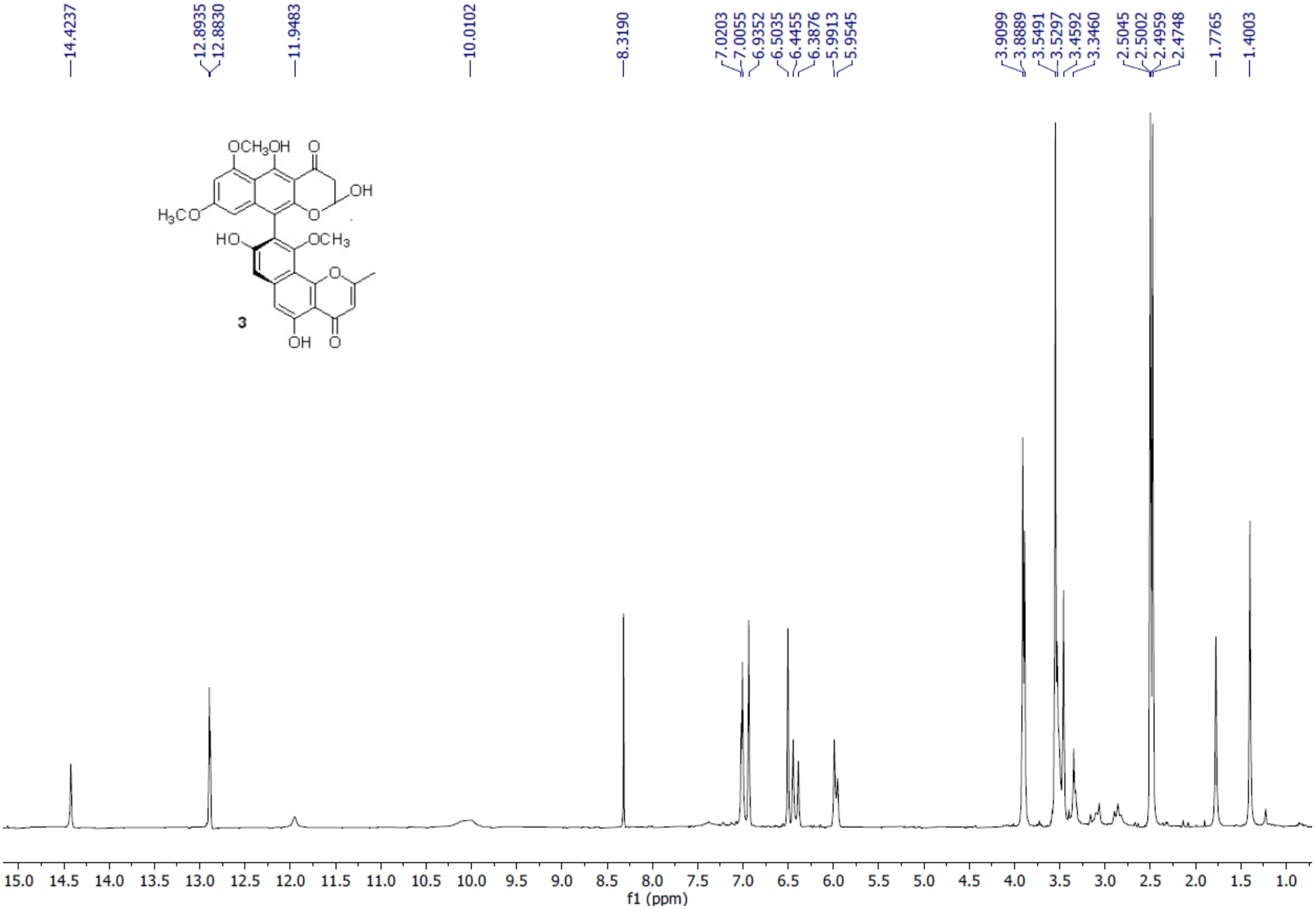

Figure S10. ^{13}C NMR Spectrum (400 MHz) of Teratopyrone C (**3**) in DMSO-d_6

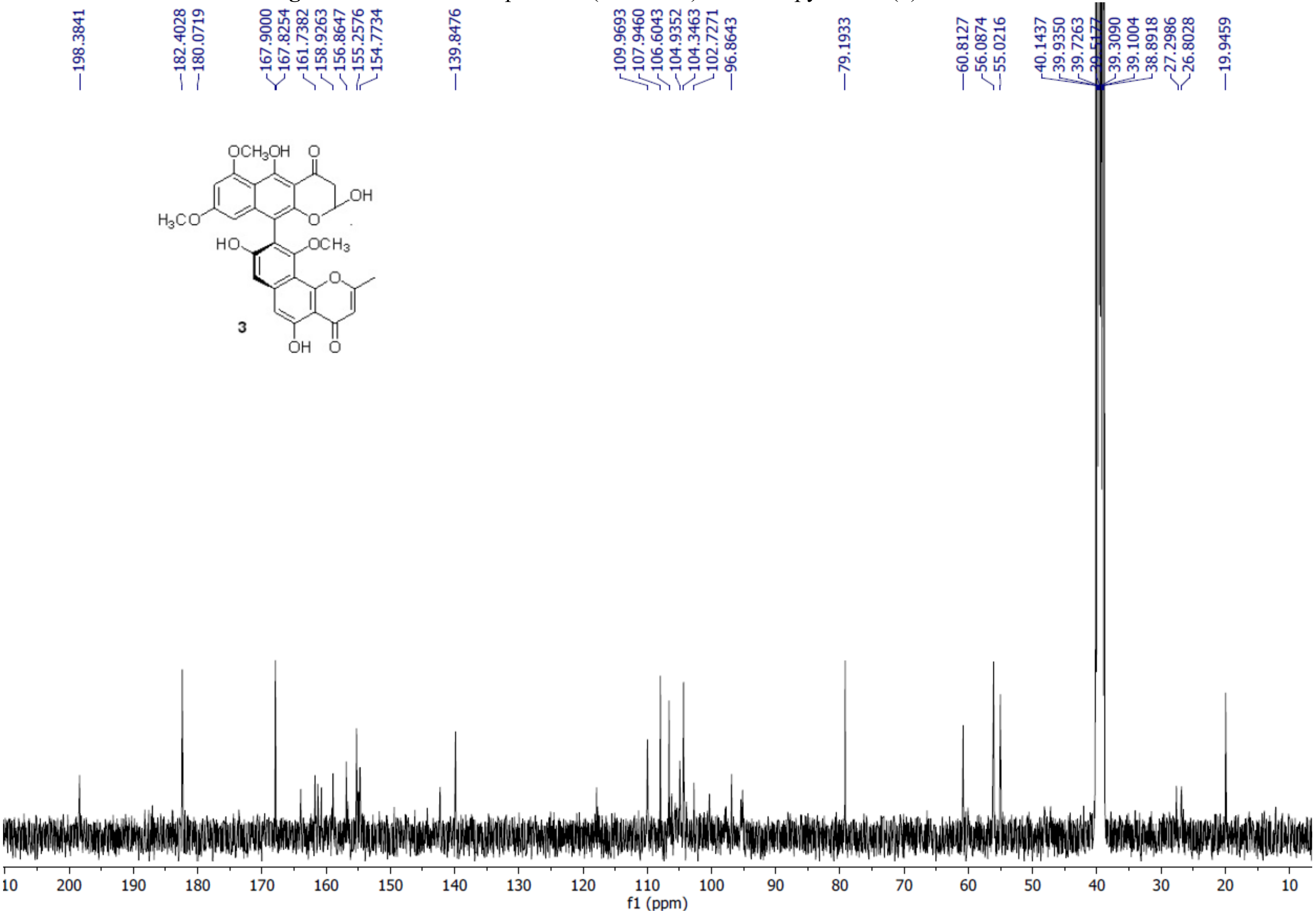

Figure S11. HSQC Spectrum (400 MHz) of Teratopyrone C (**3**) in DMSO-d_6

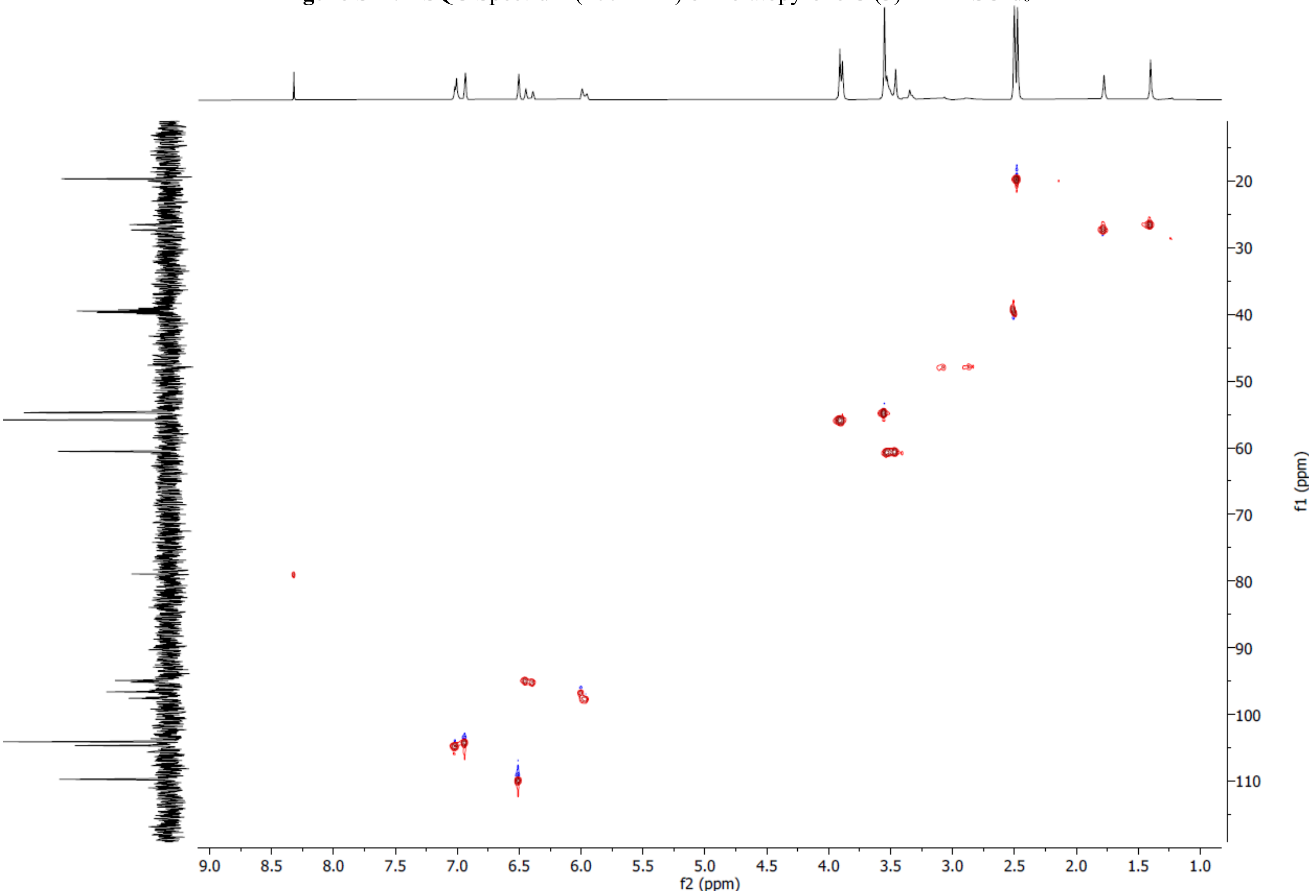

Figure S12. HMBC Spectrum (400 MHz) of Teratopyrone C (**3**) in DMSO-d6

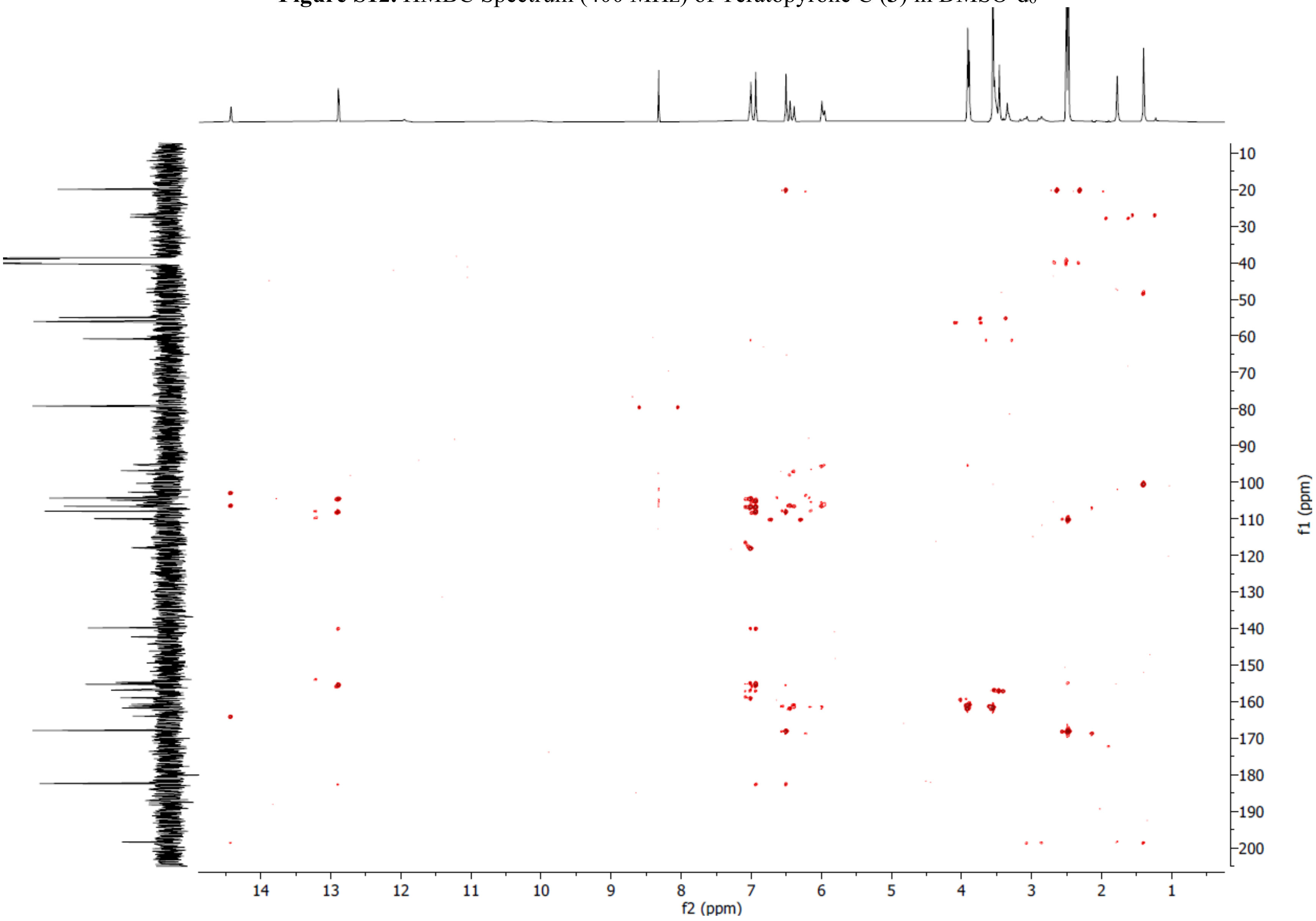

Figure S13. Results of maximum likelihood analysis placing strain AK1128 within *Teratosphaeria* with high support. Numbers in association with nodes indicate bootstrap values (values ≥ 70 are shown).

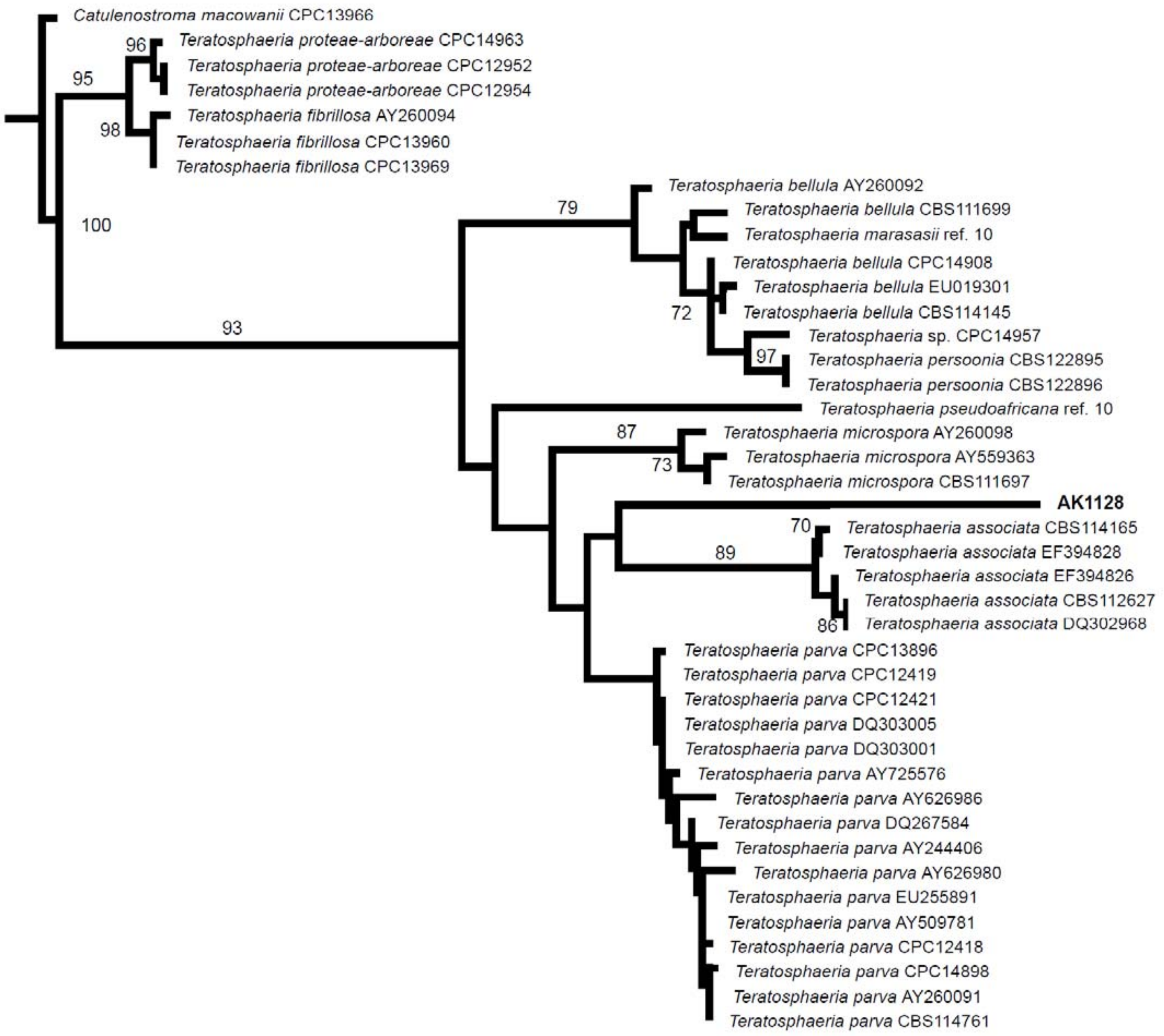

Supplement: Supplementary file 1 [file molecules-25-05058-s001.pdf]
